# Succinylcholine Administration and Resultant Pulseless Ventricular Tachycardia: A Case Report of Communication Breakdown During an Emergent Intubation

**DOI:** 10.7759/cureus.8031

**Published:** 2020-05-08

**Authors:** Jake Toy, Mark Comunale, Ho-Wang Yuen, Fanglong Dong, Michael Neeki

**Affiliations:** 1 Emergency Medicine, Arrowhead Regional Medical Center, Colton, USA; 2 Anesthesiology, Arrowhead Regional Medical Center, Colton, USA

**Keywords:** communication, patient safety, succinylcholine, guillain-barré syndrome

## Abstract

Poor communication continues to be one of the leading root causes of sentinel events in the United States annually. This case report documents a miscommunication that occurred during the management of a patient with Guillain-Barré syndrome (GBS) and acute respiratory failure requiring emergent intubation, which resulted in a transient hyperkalemia and subsequent cardiac arrest.

## Introduction

Emergency stabilization and management of critically ill patients represent one of the most challenging aspects of medical care. Stress is inherently intensified during the management of life-threatening conditions [1-2]. The heightened stress and sense of urgency in turn places an increased demand not only on a physician’s clinical skills, but also challenges their ability to maintain effective and appropriate interprofessional communication [3].

Little research exists to aid clinicians in better understanding the sources of communication breakdowns that occur during the emergent management of patients in a hospital setting. We present a case of miscommunication that occurred during the management of a patient with Guillain-Barré syndrome (GBS) and acute respiratory failure requiring emergent intubation, which resulted in a transient hyperkalemia and subsequent cardiac arrest. This case report was approved by the Institutional Review Board at Arrowhead Regional Medical Center (ARMC) and written consent was obtained from the patient for publication.

## Case presentation

The patient is a 63-year-old female who was hospitalized secondary to the development of GBS following an episode of viral enteritis. On the 18th day of her hospitalization, the patient’s vital capacity declined to 0.625 L and she developed respiratory distress. The Rapid Assessment Team (RAT) was activated and the anesthesiology department was subsequently consulted for an emergent intubation. Rapid sequence intubation was performed with etomidate 12 mg and succinylcholine 100 mg. 

Shortly after the medications were administered, the patient’s sinus rhythm deteriorated into a pulseless ventricular tachycardia. Advanced cardiac life support protocol was initiated. Within a minute of dysrhythmia, cardioversion was attempted. The rhythm then progressed to ventricular fibrillation. Calcium chloride and sodium bicarbonate were given for suspected succinylcholine-induced hyperkalemia. The patient also received calcium gluconate, amiodarone, and one additional defibrillation. Approximately 13 min after the initial rhythm change, return of normal sinus rhythm, spontaneous circulation, and neurologic function were obtained.

Serum potassium was noted to be 3.1 mEq/L six hours prior to the event. Immediate postresuscitation serum potassium was noted at 7.1 mEq/L. Follow-up analysis drawn 90 min after the event showed that potassium levels had normalized to 3.1 mEq/L. This patient was subsequently discharged home with a tracheostomy button after a 118-day hospital stay.

## Discussion

This case demonstrates a breakdown in communication at the bedside during an emergent situation, resulting in an adverse patient outcome. It is well known that severe hyperkalemia can occur following succinylcholine administration in patients with certain pathologic states such as upper or lower motor neuron injuries, demyelinating diseases, burn injuries, or massive spinal cord trauma [4-5].

In this case, the patient’s pertinent medical history was not effectively communicated between interdisciplinary provider teams. When a Code RAT activation occurs, the members of the RAT are often unfamiliar with the patient’s medical history. Upon arrival of the RAT (including a critical care registered nurse, respiratory care practitioner, and on-call medicine resident) at this patient’s bedside, the patient’s current provider team (including the unit nursing staff) began a rapid hand-off to the RAT, noting respiratory distress as the primary reason for the Code RAT activation as well as transmitting patient background information and most recent low potassium value (3.1 mEq/L). Whether this handoff included the patient’s pertinent history of GBS is unknown. However, the subsequent report to the consulting anesthesiologist did not include the GBS history. Absence of a clearly defined and structured information transfer paradigm to direct communication during this emergent medical event may have contributed to this adverse patient outcome.

Poor communication causes nearly 30% of preventable medical errors and continues to be one of the leading root causes of sentinel events in the United States annually. In a recent Joint Commission report, poor communication was associated with 489 of 764 sentinel events in 2014 (versus human factors and leadership breakdown which were associated with 547 and 517 sentinel events respectively) [6]. Indeed, handoffs have been demonstrated to be a period when communication error is most likely to occur, often due to lack of a standardized handoff and/or poor pre-handoff preparation [7]. In emergency situations, the impact of stress is likely to further exacerbate already poor communication, increasing the risk of adverse patient outcomes [1, 8]. 

Other factors that may contribute to communication breakdowns are differences in each discipline’s culture and goals for patient care, and hierarchical relationships, such as between a nurse and attending physician [7, 9]. Many facilities have adopted the SBAR (Situation, Background, Assessment, and Recommendation) tool that has been shown to facilitate improved handoff communication within care teams and improve patient outcomes in various clinical settings [10]. Benefits of SBAR include standardization of handoffs, consolidation of patient information prior to a handoff, and bridging of differences between members of varying specialties, facilitating teamwork [11]. However, SBAR can be difficult to implement as its application is nonspecific and some may view it as a document rather than a verbal communication tool [12].

**Figure 1:**
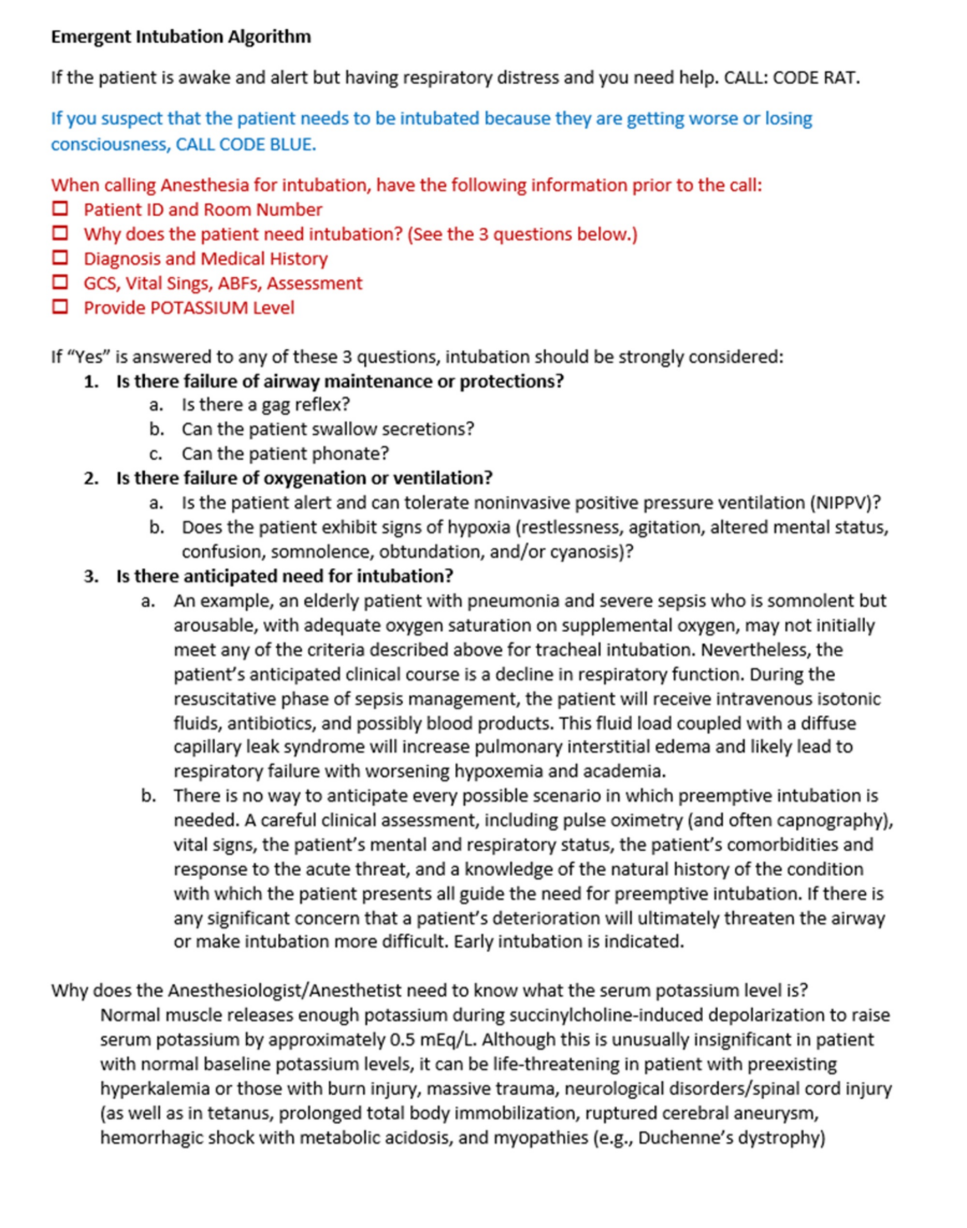
Emergent Intubation Algorithm and abridged Code RAT and Code Blue criteria. GCS, Glascow Coma Scale; ABF, Aortic blood flow; RAT, Rapid Assessment Team

An outcome of the root-cause-analysis following the presented case was the development of an Emergent Intubation Algorithm to be utilized as a guide for communication with consulting anesthesiologists at ARMC. Figure 1 presented the detailed algorithm. Utilization of this tool allows for improved communication with anesthesia teams upon their arrival by aiding in the consistent transmission of patients’ anesthesia-relevant medical history, including the most recent potassium level. Unlike SBAR, this communication tool is specific to one situation, which may increase the likelihood of compliance. 

In the seven months since implementation of the Emergent Intubation Algorithm at ARMC (December 2015 - August 2016), there have been 466 total RAT and Code Blue activations. Some 378 have been RAT activations with the vast majority occurring in telemetry units. Some 88 have been Code Blue activations primarily occurring in the ICUs, EDs, or operating rooms. Since implementation, no adverse events related to airway management during RAT or Code Blue activations have been directly associated with communication breakdowns.

## Conclusions

Despite the recognized importance of handoff communication in health care, lack of appropriate communication among providers during emergency situations continues to be a major patient safety hazard. It is understood that nothing can completely remove the stress and pressure on clinicians during emergent patient management. However, tools such as the Emergent Intubation Algorithm may have the potential to reduce variability in communication amongst providers and thus, increase the likelihood that relevant information is not left out during patient handoffs.

## References

[1] Schull MJ, Ferris LE, Tu JV, Hux JE, Redelmeier DA (2001). Problems for clinical judgement: 3. Thinking clearly in an emergency. CMAJ.

[2] Chambers R, Wall D, Campbell I (1996). Stresses, coping mechanisms and job satisfaction in general practitioner registrars. Br J Gen Pract.

[3] Xiao Y, Hunter WA, Mackenzie CF, Jefferies NJ, Horst RL, Lotas Group (1996). Task complexity in emergency medical care and its implications for team coordination. Hum Factors.

[4] Mallon W, Keim S, Shoenberger J, Walls R (2009). Rocuronium vs. succinylcholine in the emergency department: a critical appraisal. J Emerg Med.

[5] Martyn J, Richtsfeld M (2006). Succinylcholine-induced hyperkalemia in acquired pathologic states: etiologic factors and molecular mechanisms. Anesthesiology.

[6] (2016). Sentinel Event Data—Root Causes by Event Type: 2004-2015. https://www.jointcommission.org/..

[7] Abraham J, Nguyen V, Almoosa KF, Patel B, Patel VL (2011). Falling through the cracks: information breakdowns in critical care handoff communication. AMIA Annu Symp Proc.

[8] Donchin Y, Gopher D, Olin M (2003). A look into the nature and causes of human errors in the intensive care unit. Qual Saf Health Care.

[9] Sutcliffe KM, Lewton E, Rosenthal MM (2004). Communication failures: an insidious contributor to medical mishaps. Acad Med.

[10] Field TS, Tjia J, Mazor KM (2011). Randomized trial of a warfarin communication protocol for nursing homes: an SBAR-based approach. Am J Med.

[11] Leonard M, Graham S, Bonacum D (2004). The human factor: the critical importance of effective teamwork and communication in providing safe care. Qual Saf Health Care.

[12] Compton J, Copeland K, Flanders S, Cassity C, Spetman M, Xiao Y, Kennerly D (2012). Implementing SBAR across a large multihospital health system. Jt Comm J Qual Patient Saf.

